# Evolution of industrial diversification and its determinants in West Germany: Evidence from population data of enterprises

**DOI:** 10.1371/journal.pone.0259352

**Published:** 2021-11-03

**Authors:** Sandra Kublina, Muhammad Ali

**Affiliations:** 1 Friedrich Schiller University, Jena, Germany; 2 Department of Economics, School of Social Sciences and Humanities, National University of Sciences and Technology, Islamabad, Pakistan; Institute for Advanced Sustainability Studies, GERMANY

## Abstract

Germany is among the largest countries in the world in terms of total GDP, owing largely to rapid industrialization and expansion of economic activities into several sectors. This paper contributes to the literature on German economic development by investigating the evolution of industry diversification in Germany; particularly focusing on the recent concepts of related (RV) and unrelated variety (UV) in West German regions. It also identifies the statistical and economic determinants of variation in variety over time. Among several industry structure measures; RV is the only measure that reveals a pronounced increasing trend. Since RV is composed of two parts: 1) entropy at five-digit within a two-digit classification, and 2) shares of two-digit sectors in total output, we examined which of the two components dominate. Our findings suggest that the entropy component within two-digit sectoral shares of the RV index is more dominant than the two-digit sectoral shares themselves. We further examined entries and exits of the firms among regions with top and bottom rankings in RV. Findings suggest that both the top and bottom regions experienced an increase in the total number of industries, however, exits were much less pronounced in the bottom regions. It suggests that an increase in variety among top regions is the result of the creative destruction type effect where new industries force inefficient old industries to leave the region. Finally, analysis shows support for the inverse u-shaped relationship between development and diversification.

## Introduction

Regional growth literature identifies the industrial composition of regions as one of the key determinants of growth. Some studies argue that specialization is beneficial for growth while other studies show that diversification allows regions to follow a sustainable growth trajectory [1–3]. Recent evidence also suggests that diversification and specialization strategies vary at different stages of development [4]. Despite contradictory evidence, stylized facts seem to favor diversification rather than specialization for economic development as diversified economic structure provides resistance against external shocks and results in radical innovation through recombination of knowledge across sectors [5–7]. The importance of regional industrial structure for recombination of knowledge arises from the fact that that some knowledge spillovers are sticky to their locations due to the tacit nature of knowledge. Therefore, the most efficient way to transfer tacit knowledge is through face-to-face contact [8].

There are two contradictory theories on how knowledge spillovers take place in a region, namely, Marshall-Arrow-Romer (MAR) externalities and Jacobs externalities. MAR externalities suggest that knowledge is sector-specific; hence, specialization accelerates knowledge spillovers within industries which accelerates economic growth. On the contrary [6], argued that a diversified industrial structure allows the recombination of knowledge across industries. Such knowledge transfers are more likely to result in radical types of innovations as compared to incremental innovations in the case of specialization [9]. Alternatively, diversity can also be viewed in terms of the portfolio theory in which an increase in diversity reduces the vulnerability of regions to external shocks [10].

Diversification can be disentangled into related and unrelated variety as proposed by [9]. Authors propose that knowledge spillovers are more frequent among related industries as compared to unrelated ones as cognitive proximity of related industries facilitates knowledge spillovers. While the impact of related and unrelated variety on regional growth has been widely empirically examined in recent years, much less is known about how the patterns of diversification evolve and what determines a certain evolution pattern.

According to the World Development indicators, Germany is one of the most industrialized countries in the G7 in terms of industrial value-added as a percentage of GDP. It is the largest economy in the EU with its GDP standing at 3.85 trillion USD in 2019 (World Development Indicators (WDI)). Despite the global significance of the German economy, the literature on the evolution of sectoral composition in Germany especially approached from the related and unrelated variety perspective, is scarce. Therefore, this study investigates long-term development patterns of regional industrial diversity, related variety (RV), and unrelated variety (UV). The unique feature of this study is that it uses the data for entire population of enterprises in West German regions over more than thirty years from 1976 until 2010. The data is accessed through special permission from IAB—Institut für Arbeitsmarkt- und Berufsforschung der Bundesagentur für Arbeit in Nurnberg, Germany. The paper deals with the following main research questions:

- How has industrial structure emerged in West Germany over time? Has there been any difference in the evolution of diversification patterns in related and unrelated sectors?
- What is the driving force behind strong increasing trends of related variety in West Germany over time? Two sources are addressed: First, is it driven by sectoral shares of two-digit industries or entropy within two-digit industries? Second, the role of sectoral entries and exits on the increase in RV is investigated.
- Do diversification patterns vary at different stages of regional development and depending on proximity to highly diversified regions?

The paper is organized as follows. Section 2 provides an overview of the main theoretical concepts related to industrial composition and their associated different types of externalities. Data, spatial framework, and construction of indices are outlined in Section 3. Section 4 provides an overview of the long-term development patterns of industry structure in West Germany and further differentiates between related and unrelated diversification. Section 5 analyzes statistical and economic drivers of the strong increasing trend in RV in West Germany. Section 6 concludes by indicating some future research directions on this topic.

## Literature review

The role of industrial composition in the diffusion of knowledge across sectors has been recognized and debated since [11]. One strand of the literature proposes that specialization in few industries ensures learning-by-doing effects, and knowledge transfers are relatively frequent under such a structure. Externalities arising from similar sectors are termed as “localization externalities”, or MAR externalities, named after decades of reformulation of ideas by [11–13]. Specialized industrial composition benefits from specialized labor, specialized suppliers, and large markets. On the contrary [6], argued that externalities associated with diversified industry structures are stronger determinants of long-run regional growth as compared to localization externalities. According to this point of view, diversity provides opportunities to interact and recombine practices and thus could foster the generation of new ideas and innovations [14]. The recombination of knowledge across sectors under a diversified industrial structure is expected to lead to radical types of innovations [9]. Such positive externalities are generally termed as “Jacobs externalities”.

However, there may be significant differences in the types of innovations generated by spillovers between related and unrelated sectors. Jacobs externalities are criticized in literature due to the oversimplification of the concept. This question has been elaborated by [9] by introducing the concept of related and unrelated variety. The concepts of related and unrelated variety, proposed by [9], disentangles diversification based on the degree of relatedness among sectors to differentiate between knowledge spillovers and portfolio effects of diversification. In particular, [9] claimed that knowledge spillovers are relatively frequent among related sectors as compared to unrelated sectors. Moreover, diversification in unrelated sectors helps to protect regions from sector-specific shocks due to limited reliance on a few industries. Therefore, economic effects associated with different types of variety are expected to be different and, in the analysis of regional performance, they should be empirically separated from each other. The terms ‘diversity’ and ‘variety’ are used interchangeably in this study, although, variety is more attributed to the concept that distinguishes between related and unrelated variety. Frenken [15] point to their preference for the term ‘variety’ since ‘diversity’, according to their view, is more attributable to the biology. Similarly, ‘sectors’ and ‘industry’ are used interchangeably in this study. Spillovers between similar industries, according to [16], are more likely to lead to incremental innovation, but spillovers between unrelated industries are more likely to lead to radical innovation, such as entirely new products. If radical innovation generates more positive regional development impulses than incremental innovation, then it is unclear which form of diversity will be more significant for growth: radical or incremental. Spillovers between similar industries may be more common but have a minor impact, whereas spillovers between unrelated industries should be less common but have a larger impact [17].

Table 1 summarizes the types of externalities and innovations generally associated with specialized and diversified economic structures as explained above.

**Table 1:** Types of externalities associated with economics structures.

	Specialized industry structure	Diversified industry structure	
Types of externalities [6, 11]	MAR externalities	Jacobs externalities	
Agglomeration externalities [18]	Localization externalities	Urbanization externalities	
Sources and ways of knowledge transfer	Intra-industry learning	Inter-industry learning	
Extension of diversity concept [9]		Related variety ↓	Unrelated variety ↓
Possible growth drivers [17]	Incremental innovation	Incremental innovation	Radical innovation

Source: authors

## Data and methodology

### Data description

This study is based on a comprehensive dataset on German firms. The case of Germany is interesting because is the most industrialized country in G7 with industrial value-added as a percentage of GDP standing at 26.8% in 2019 (WDI). It is the 4^th^ most developed country according to the Human Development Index 2018 and it has the highest trade to GDP ratio in G7. It has the 3^rd^ highest GDP in PPP terms among G7 countries. Finally, it is the 8^th^ most diversified country in the EU according to the report by the European Union entitled “Competing in Global Value Chains: EU Industrial Structure Report 2013”.

This section describes the data and construction of industry representative diversification indices. The spatial framework of the analysis is the 71 planning regions of West Germany. Planning regions are functional spatial units that consist of at least one core city and the surrounding area and are comparable to the labor market areas in the United States. The choice of planning regions over districts is justified by the fact that various effects (e.g., knowledge spillovers that are of the particular importance in the concept of RV and UV) which might be relevant for larger units of an observation than districts and therefore could decrease the potential presence of spatial autocorrelation in the empirical estimations. Furthermore, labor market regions are the most appropriate spatial unit of analysis for agglomeration research [15].

The analysis is restricted to West Germany because many empirical studies indicate that the East German economy in the 1990s was a special case with very specific conditions that cannot be directly compared to those of West Germany [19]. Besides, long term data (before the 1990s) is not available for East Germany. Data on industry composition is obtained from the German Social Insurance Statistics (IAB—Institut für Arbeitsmarkt- und Berufsforschung der Bundesagentur für Arbeit). This dataset contains every establishment in Germany that employs at least one person obliged to make social insurance contributions [20]. Each establishment can be assigned to a 3-digit-level industry classification over the period covered in the analysis. All industry related measures account for changes in the industry classification over time (for details see [21]). The public sector, agriculture, and mining industries are excluded from the analysis.

### Measuring diversification

The literature on economic diversification provides a wide range of indices to capture industry structure. Depending on the assumptions behind each measure, the interpretation of diversification can be different. For discussions on diversification and specialization indices with their advantages and disadvantages see [22] or [23]. Broadly, diversification indices can be divided into absolute and relative measures depending on the benchmark used. In the context of this study, for absolute measures, the reference level is an equal distribution of employment across all industries, whereas, for relative measures, it is the average economic structure of the reference level, e.g., a country under study. Hence, relative measures reveal how dissimilar the sectoral composition of a region is compared to the reference level (which is generally a country if the unit of analysis is a region). An advantage of relative measures is that they compute a measure for a region relative to the country which minimizes the chances of over or underrepresentation of the regional industry composition if measured in isolation. Another important aspect of industry assessment is the choice of the industry aggregation level that can produce diverse empirical evidence [23].

Absolute diversification can be measured by the Theil [24] index which, in principle, is an entropy measure. Diversification measured by entropy index measures the degree to which the employment is equally distributed across sectors. When one or a few dominant industry sectors are present, the diversification index takes a low value indicating the specialization of a region in a few sectors. On the contrary, if employment is equally spread across all sectors, then the index takes its highest value indicating a high level of diversification. Being based on entropy measures, the Theil index can be decomposed at different levels of industrial classification, a property that is applied to construct the measures of related and unrelated variety below.

Relative diversification, on the other hand, is measured by the inverse of a Hirshman-Herfindahl index [25, 26] as applied by [5, 27]. The index increases with the increase in regional sectoral diversity which mirrors sectoral diversity at a national level. Relative diversification indices are not directly decomposable into the related and unrelated variety.

In what follows, the study focuses on absolute diversity measure (denoted as ‘Overall variety’) which is also decomposed into RV and UV. *Overall variety* (OV) is calculated using the entropy measure at the three-digit industry classification level in the following way:

$$Overall\;Variety\;(OV)=\sum_{i=1}^{n}p_{i}{log}_{2}(\frac{1}{p_{i}})$$

where p_i_ is employment in a three-digit industry share. The values of OV can vary from 0, when all employment is concentrated in only one three-digit sector, up to log_2_(n) when all sectors employ an equal number of employees. Data used in this study contains a total of 174 three-digit private sectors which means that the theoretical upper bound of the OV index is 7.44. Further, we distinguish between UV and RV following the methodology used by [9] who apply the decomposable nature of an entropy measure to distinguish between the related and unrelated variety.

*Unrelated variety* measures entropy across two-digit industries and thus assumes that industries at this level of aggregation are unrelated to each other because they are not cognitively proximate. A common belief is that knowledge spillovers are less frequent among unrelated sectors, however, recombination of knowledge among unrelated sectors is expected to result in radical innovations [17]. UV is calculated using the following formula:

$$Unrelated\;Variety\;(UV)=\sum_{g=1}^{G}P_{g}{log}_{2}(\frac{1}{P_{g}})$$

where P_g_ is the share of employment in the two-digit sector S_g_ (where g = 1,…,G) over the total employment in a region. The UV measures the degree to which employment shares are evenly distributed across unrelated (in this case two-digit) sectors. The values of UV can vary from 0, when all employment is concentrated in only one two-digit sector, up to log_2_(G) when all sectors employ an equal number of employees. In this study, there are a total of 41 two-digit private sectors that correspond to the theoretical upper bound of 5.36.

*Related variety* is measured as the weighted sum of entropy at the three-digit level within each two-digit sector assuming that industries within this level of aggregation are related based on cognitive proximity and thus can effectively learn from each other [9]. RV is calculated using the following formula:

$$Related\;Variety\;(RV)=\sum_{g=1}^{G}P_{g}H_{g}\;\mathrm{where}\;H_{g}=\sum_{\begin{array}{c} {i=1;S}_{i}\epsilon S_{g} \end{array}}^{I}\frac{p_{i}}{P_{g}}{log}_{2}(\frac{1}{p_{i}/P_{g}})$$

where P_g_ is the share of employment in the two-digit sector S_g_ over the total employment in a region; p_i_ is the share of employment in the three-digit sector S_i_ (where i = 1,…,I) belonging to the same two-digit sector S_g_. The RV indicates the degree to which employment at the two-digit level is evenly spread across its three-digit subsectors. The values of RV can differ from 0 when, within each two-digit sector, employment is concentrated in only one of its three-digit subsectors up to log2(I)-log2(G) when all subsectors employ an equal number of employees (based on entropy decomposition theorem by [24], as applied by [17]). The higher the RV value is, the more evenly employment is spread across the subsectors indicating to the higher number of technologically related industries in a region. According to the underlying concept of the RV, such industry structure is conducive to inter-sectoral knowledge spillovers. In this study, there are a total of 174 three-digit subsectors (I) under 41 two-digit sectors (G) which means that the theoretical upper bound of the index is 2.09.

## Results

This section presents the findings of the study in two parts. The first part presents the trends of diversification over time and the second part presents the determinants of diversification.

### Trends of overall, related and unrelated diversification over time

#### Evolution of overall industrial structure over time

**Fig 1:**
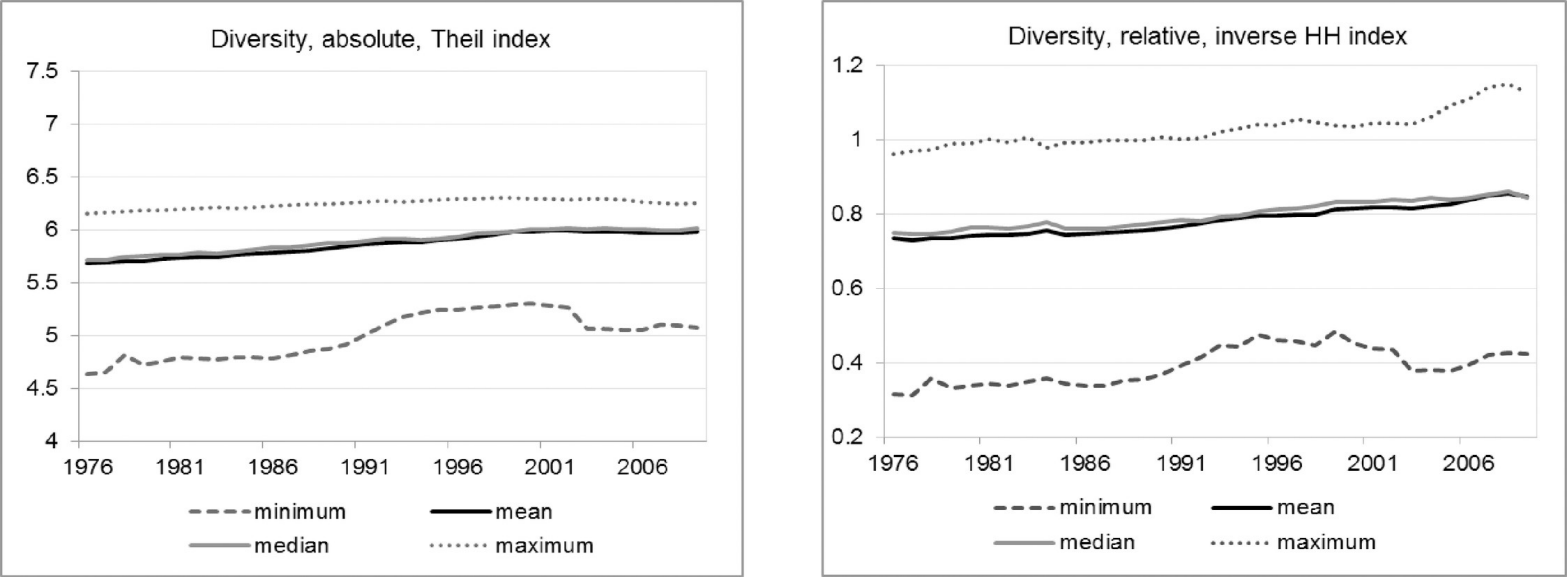
Evolution of absolute and relative diversity over time. Source: Authors.

The evolution of absolute industry diversification measured by Theil index and relative diversification measured by the inverse HH index are presented in Fig 1. For each year, the dashed line represents the region with the lowest diversification whereas the dotted line shows the region with the maximum diversification. The black solid line represents the mean value whereas the grey solid line represents the median value of diversification. The absolute diversification index shows relatively less variation as compared to the relative one. The solid lines for the mean and median of the diversification indices show that there has been a slight increase in diversification over 34 years. In particular, mean absolute diversification has increased by 5% (0.1% annual growth on average) whereas mean relative diversification has increased by 15% (0.4% annual growth on average). Overall, Fig 1 shows that industrial diversification in West Germany has increased but quite slowly over 34 years. However, it should be noted that these indices are measured at a high level of aggregation due to which, some inter-sectoral dynamics might be suppressed within the indices. For this reason, the overall variety measure is decomposed into its related and unrelated components which are further analyzed in the Sections 0–0.

#### Evolution trends of related and unrelated variety over time

This section disentangles overall variety into the related and unrelated variety and compares these variety measures with general evolution in industry structure. Long term patterns of industry structure shown in Section 0, in line with other studies on developed economies, reveal slow growth in diversification. On the evolution of RV and UV [14], based on findings of [28, 29], conclude that expansion in unrelated sectors is likely to be persistent over time because it is more likely for regions to diversify in activities that are related to existing fields. Also [30], note that existing evidence shows that new industries do not appear out of the blue, rather they evolve from existing industries and competencies that are available in regions.

**Fig 2:**
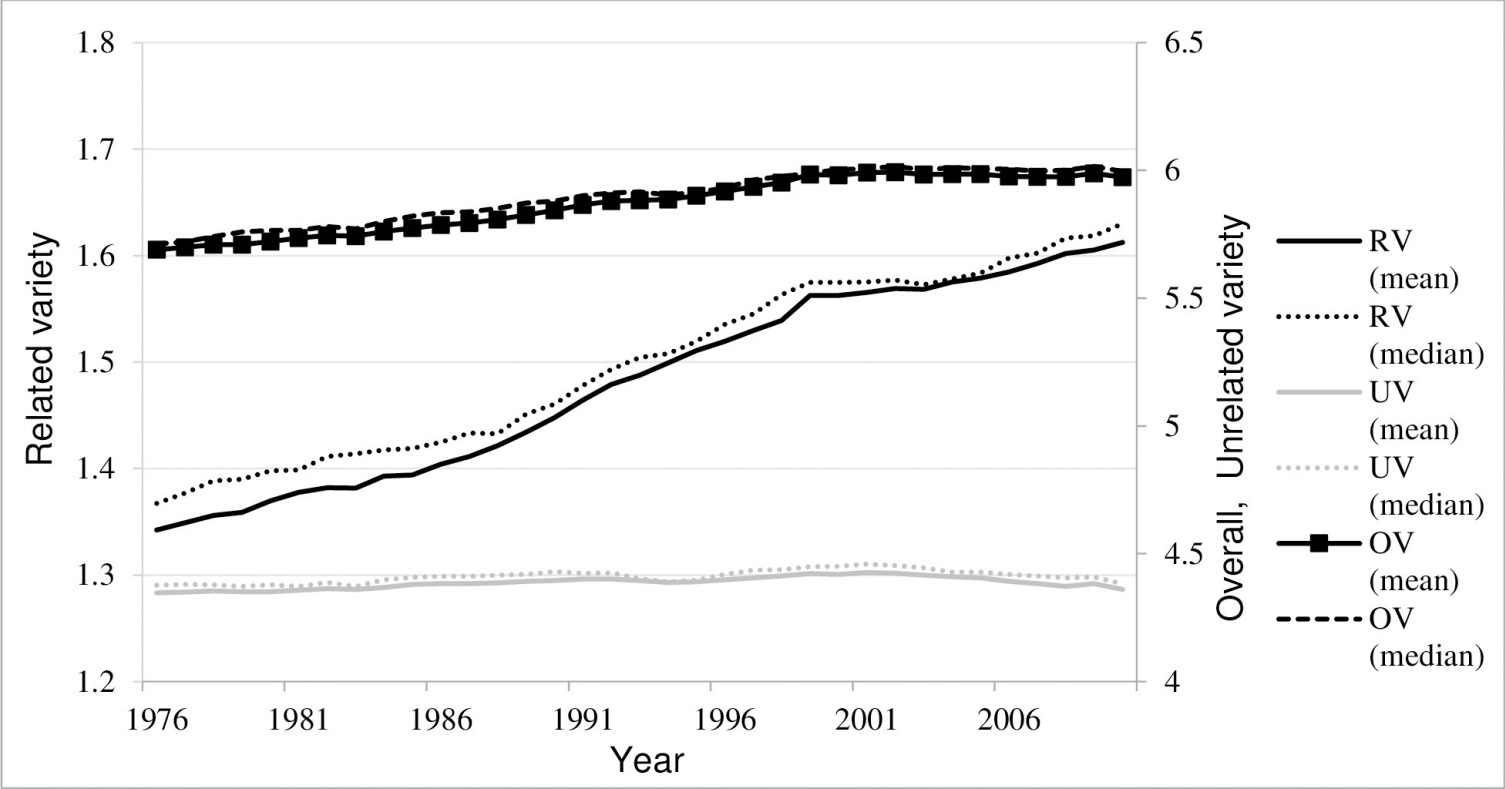
Development of overall, related and unrelated variety over time. Source: Authors.

**Fig 3:**
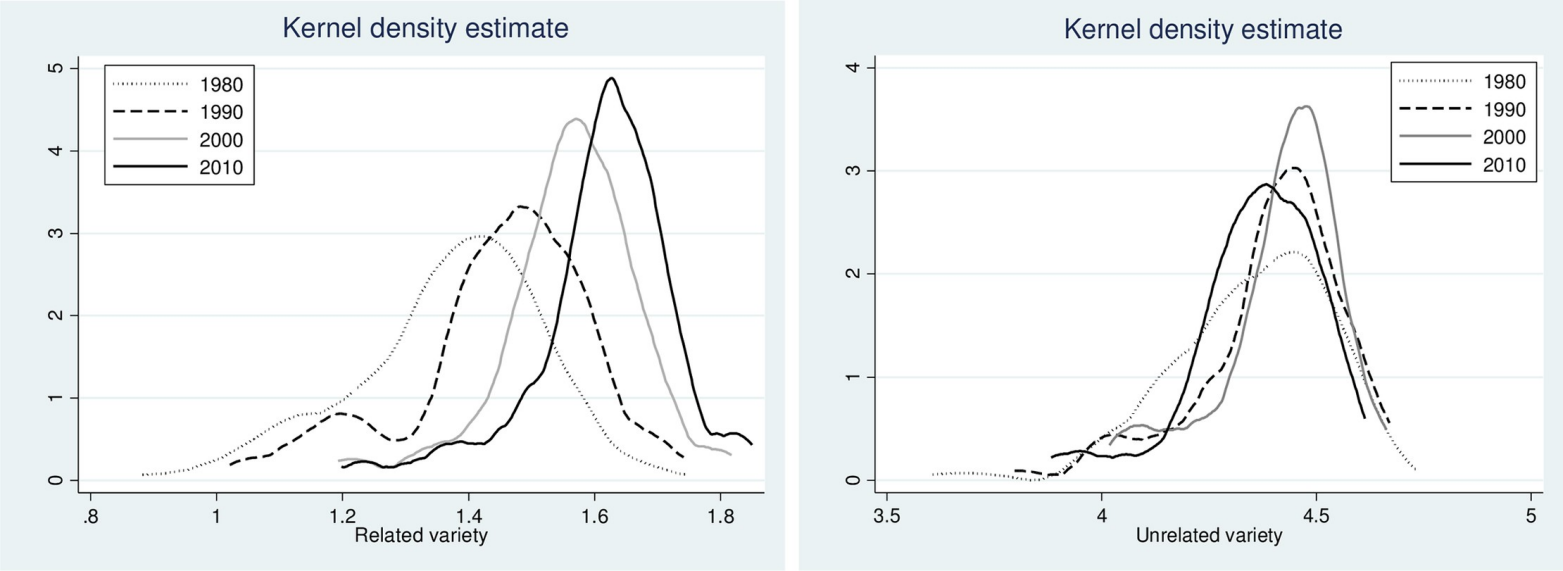
Probability density for related and unrelated variety. Source: Authors.

Long term development patterns of OV and its decomposition into RV and UV is shown in Fig 2. The average values of UV over the years of observation lie between 4.35–4.42. Considering the theoretical upper bound of UV being 5.39, this indicates a rather diverse yet stable unrelated industry composition of West German regions. Such high stability might be explained by difficulties to attract new industries if they are not technologically proximate to current regional activities (as shown by [31]). While UV remains largely unchanged over time, RV reveals a continuous increase for West German regions. The average values of RV have increased over the years of observation from 1.34 up to 1.61. Fig 3 indicates that the RV component of the OV is the driving force behind the slightly increasing trend of the OV. An increase in RV means that the employment distribution across three-digit sectors within each two-digit class is becoming more evenly spread. Theoretically, there can be three possible technical reasons for changes in RV: 1) changes in sectoral shares with no change in total sectors, 2) entry of new sector(s) and 3) exits of the old sector(s). A brief analysis of these possible dynamics is presented in Section 0.

Regarding the evolution of RV over time, there is almost no evidence for long term RV trends in the literature to make a reasonable comparison between West Germany and other economies. The study by [32] is an exception which, for the period between 1993 and 2006, reveals a similar trend of the continuous increase in RV for Finnish regions. Reasons for such a trend, however, are unclear and have not been analyzed in detail. Nevertheless, the particular period in Finland is described as the one during which the Finnish economy experienced a shift to a high tech economy, and the relevance of intra-industry knowledge spillovers for high tech sectors is emphasized [32]. From the theoretical perspective, it might mean that the development of the high-tech sectors has primarily taken place via incremental type innovation.

Continuous increase in RV might be viewed in the context of those few studies that show evidence that regions tend to expand and diversify in activities that are related to those of existing ones. By exploring long term development patterns of Swedish regions from 1969 to 2002 [31], showed that regions are more likely to expand in industries that are related to their existing industry portfolio. Furthermore, even if the newly introduced industries are conceptually related to each other, they might still be different from the existing portfolio of industries present in the region and may result in increasing related variety. Boschma [30] investigated a similar question for Spanish regions from 1988 to 2007 and concluded that new industries that emerged in Spanish regions tended to use similar capabilities of existing industries. It is important to note that these studies apply different relatedness indicators to measure regional related diversification. Neffke [31] measured relatedness based on co-occurrence of products from diverse industries within portfolios of manufacturing plants, whereas [31] used a product proximity indicator developed by [30] to determine to what extent two products share similar capabilities.

Looking at the evolution of related and unrelated variety from a different lens, the analysis of the probability density function over time (Fig 3) reveals another interesting trend for RV. In addition to the shift of the RV distribution to the right side, which reflects an increase in absolute values, there is also a pronounced change in the shape of the distribution. Distribution of the RV gets steeper over time indicating that regions converged around a mean variety in related activities. Distribution of UV confirms long term persistence in absolute values relative to related variety. There is also a change in the shape of the distribution, however, it is less pronounced and consistent compared to RV.

#### Persistence of related and unrelated variety over time

**Fig 4:**
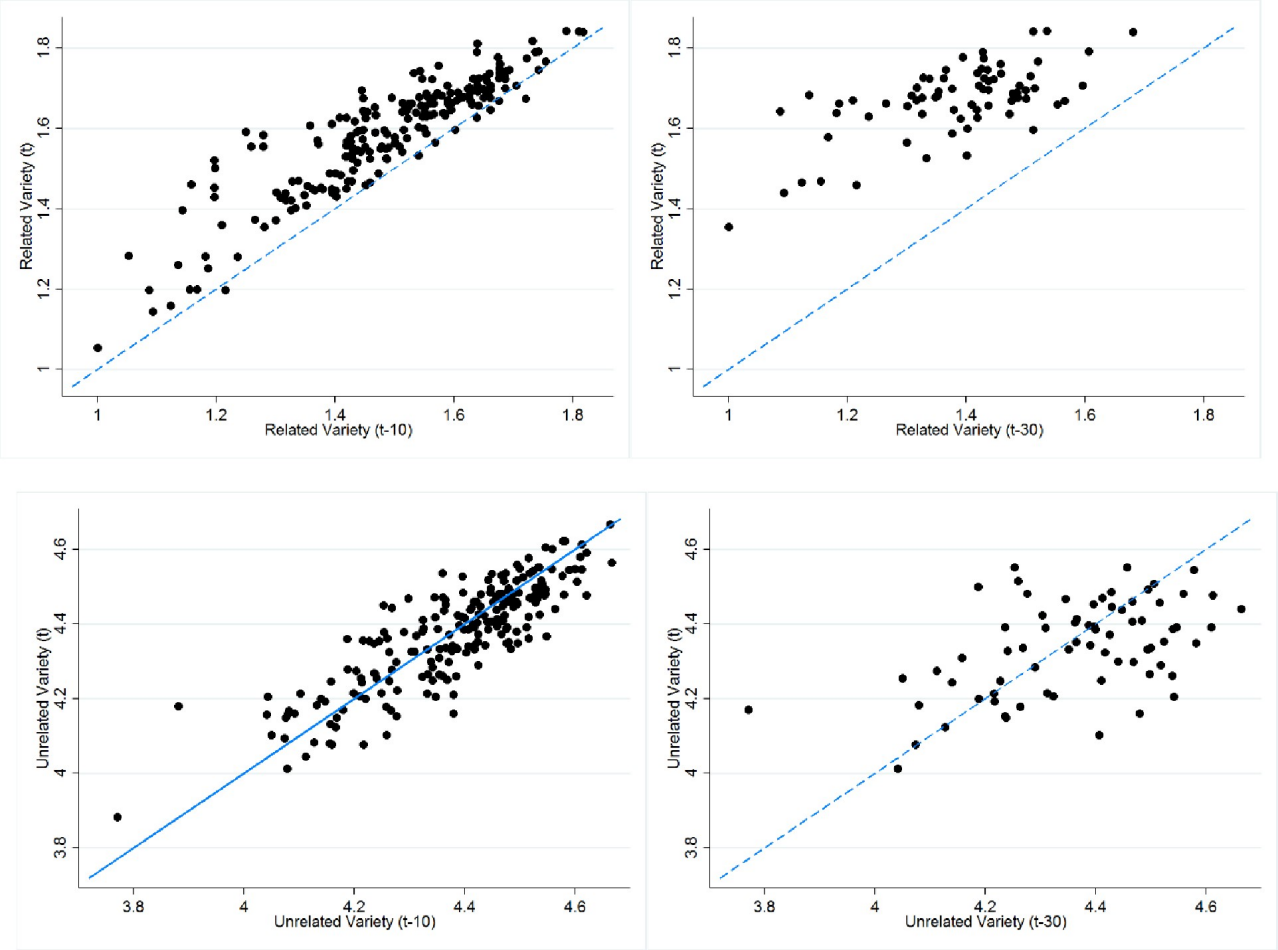
**Relationship between related variety in period t and t-10 (on the left) and t and t-30 (on the right).** Source: Authors.

Fig 4 illustrates the range of variation and the level of persistence of variety indices relative to its state ten and thirty years ago. In these graphs, the 45-degree line indicates no change in variety over time. Fig 4 show that regional related variety increases over time as most of the regions lie above the 45-degree line. This is especially pronounced when the current variety is compared with its level thirty years ago. Changes over time confirm previously described an increase in the overall RV for planning regions. Contrary to RV, the evolution of UV is less pronounced as most of the regions are spread around the 45-degree line showing low or no change over ten and thirty years.

Munich is among the top 5 regions with the most unrelated industrial structure in all years covered in this study and it is known for its vibrant entrepreneurial spirit. Other regions include Unterer Neckar and Mittlerer Oberrhein, which share borders with Munich, and Aachen and Göttingen are among regions with high unrelated variety. In nearly all years of analysis, the lowest UV is observed for Ingolstadt which is a host of the giant automobile producer Audi (other regions with the lowest UV in all years of the analysis are Main-Rhön, Braunschweig, Landshut, and Siegen). Among the top 5 regions with the most related industrial structure are Düsseldorf; Bochum/Hagen; Duisburg/Essen; Hamburg and Siegen. It is interesting to note that 4 out of the bottom 5 regions with the lowest average RV are the same as the bottom UV positions–these are Ingolstadt, Braunschweig, Main-Rhön, Landshut, and Rheinpfalz.

#### Spatial persistence of related and unrelated variety

**Fig 5:**
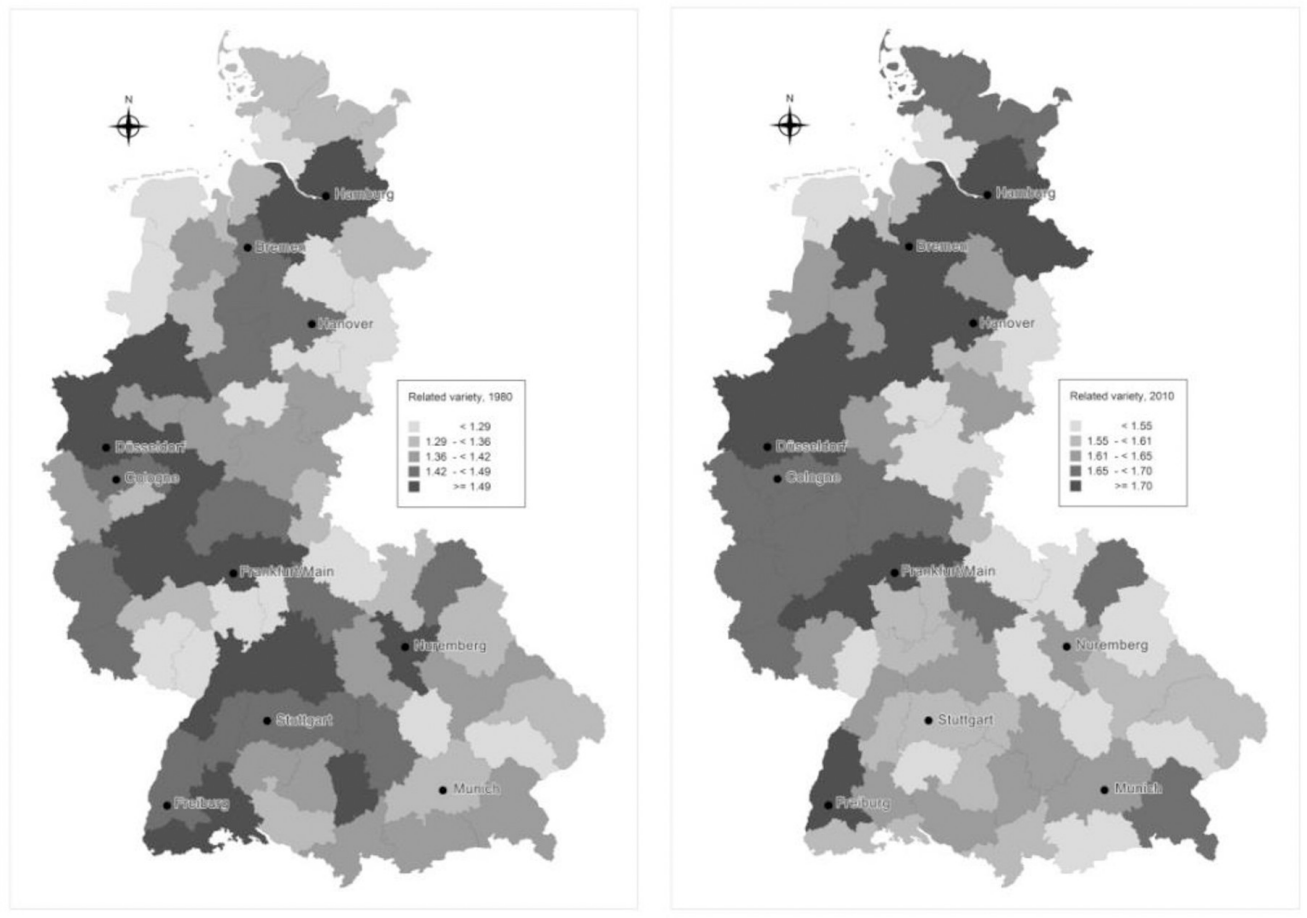
**Related variety in 1976 (on the left) and 2010 (on the right).** Source: Authors.

**Fig 6:**
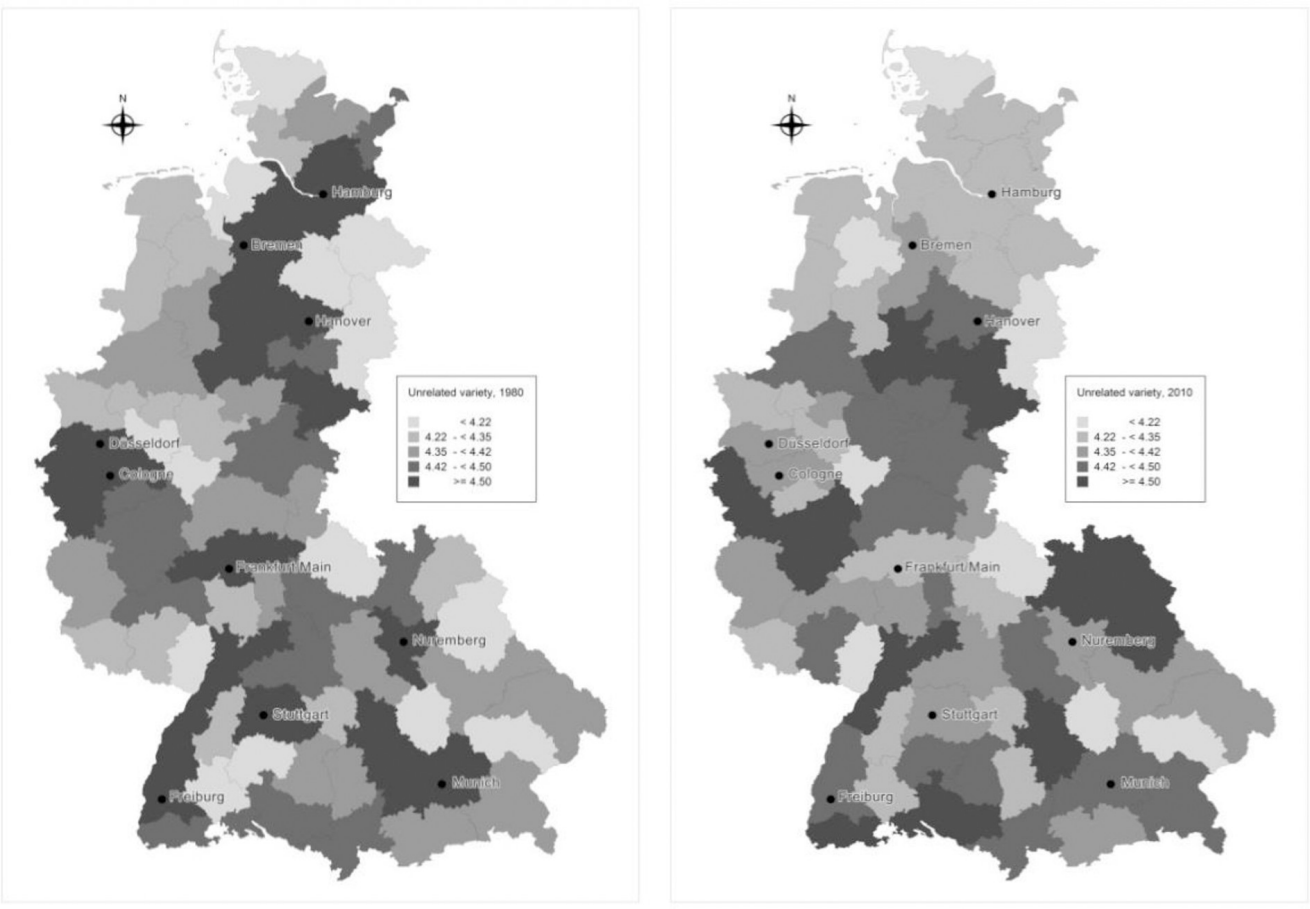
**Unrelated variety in 1976 (on the left) and 2010 (on the right).** Source: Authors.

Spatial distribution of the variety measures across districts for the beginning (1976) and the end of the analysis (2010) is displayed in Figs 5 and 6. Apart from the continuous increase in value and change in distribution, RV also reveals a change in spatial distribution. In 1976, high values of RV are rather evenly distributed across space, whereas, in 2010 they tend to localize in Central and North Western regions forming a cluster in the central regions. Another pronounced trend is the decrease in relative RV positions for southern regions. Exploration of causes for changes in the spatial distribution of RV is an issue for further research. Concerning the spatial distribution of UV, high values seem to be rather evenly distributed across space. With regards to the development trends over time, a slight change is observed for the North regions which decreased in relative positions of UV.

### Statistical and economic determinants of variation in variety indices

This section analyzes the driving forces behind the continuous increasing trend of one type of variety, namely related variety, in West Germany over time. First, it studies the extent to which such an increase in RV is driven by sectoral shares of two-digit industries or entropy within two-digit industries (Section 0). Second, it studies the role of entries and exits of sectors in the industrial composition on the increase in RV (Section 0). Finally, Section 0 estimates the general relationship between both types of varieties and regional development as well as the distance to variety frontiers.

#### The effect of changes in sectoral shares (Pg) and entropy within these sectors (Hg) on the evolution of the related variety

As shown in Section 0, Related Variety is a weighted sum of entropy at the three-digit level within each two-digit sector. Entropy within each two-digit sector (Hg) is weighted by the share of the two-digit sector (Pg) in total employment of the region. The intuition behind assigning weights is that the measure of entropy is unaffected by the magnitude of employment. As a result, even though sectors with significantly different levels of employment are likely to have very different effects on the overall economy, they still can have the same entropy levels. Therefore, entropy within two-digit sectors is weighted by sectoral shares to give more weight to the sectors with larger shares in total employment.

The interpretation of the increase in RV is generally attributed to the increase in sectoral shares of industries related to each other. This interpretation implies that the increase in RV is driven by entropy within two-digit sectors (Hg). However, since measurement of RV involves Pg and Hg, the variation in the index can be amplified by either two-digit sectoral shares (Pg), entropy within these sectors (Hg), or both. If the effect of Pg is dominant, then the interpretation of the increase in RV becomes less trivial as one can no longer associate the increase in RV with the increase in entropy in related sectors.

**Fig 7:**
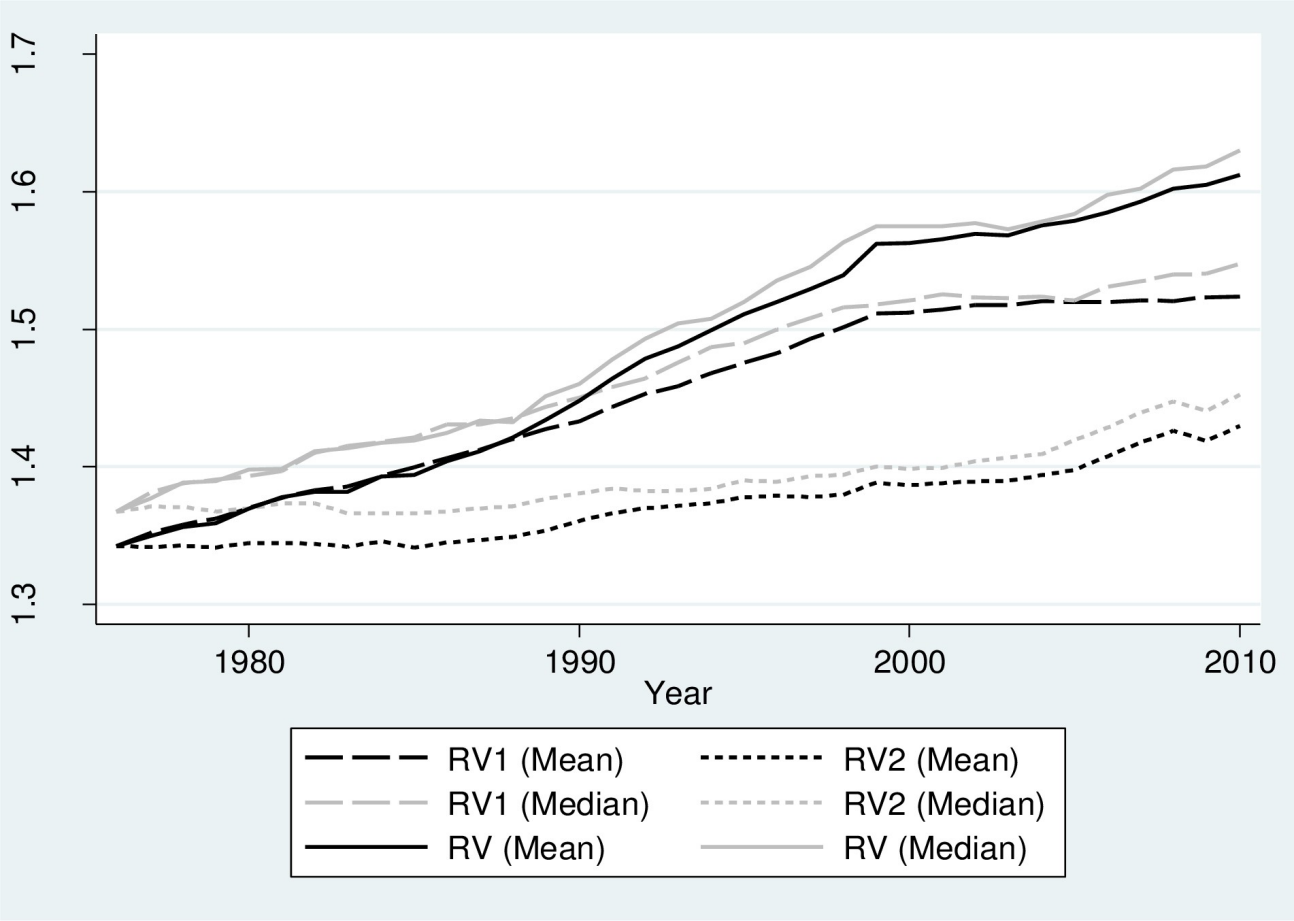
Relative importance of Pg and Hg in the growth of RV over time. Notes: RV1: Related variety measured by keeping Pg constant at the values at 1976. RV2: Related variety measured by keeping Hg constant at the values at 1976. Dashed lines represent median values of RV across regions.

To analyze whether the strong increasing trend in RV in West Germany is driven by Pg or Hg, two additional RV indices are introduced in the analysis. In the first index (RV1), Pg is held constant at its level in 1976, while Hg can vary. In practice, such transformation would imply that, while sectoral shares can vary at the three-digit level, they do not affect the broad industry structure at two-digit levels. Likewise, in the second index (RV2), Hg is held constant at its level in 1976, while Pg can vary. In this case. industry distribution at its lowest (three-digit) level is held constant, while aggregated industry structure at the two-digit level can vary over time. Fig 7 compares the trend of the original RV index with two artificially generated indices RV1 and RV2. The indices are calculated for West Germany by using mean (black lines) and median (grey lines) across regions. Fig 7 shows that RV and RV1 are overlapping from 1976 to 1989, and RV1 shows a strong increasing trend in the later years. On the contrary, the line plot for RV2 shows, on average, a relatively weaker positive trend especially until 1990. Fig 7 shows that the trend in RV is mostly driven by an increase in Hg. Therefore, it can be concluded that the increase in RV in West German regions has taken place via the increase in variety in related sectors rather than the increase in shares at a broader (two-digit) sectoral level. In other words, the driving force behind the increase in RV is a change in the distribution of sectoral shares within two-digit sectors rather than the magnitude of employment at the two-digit level.

**Fig 8:**
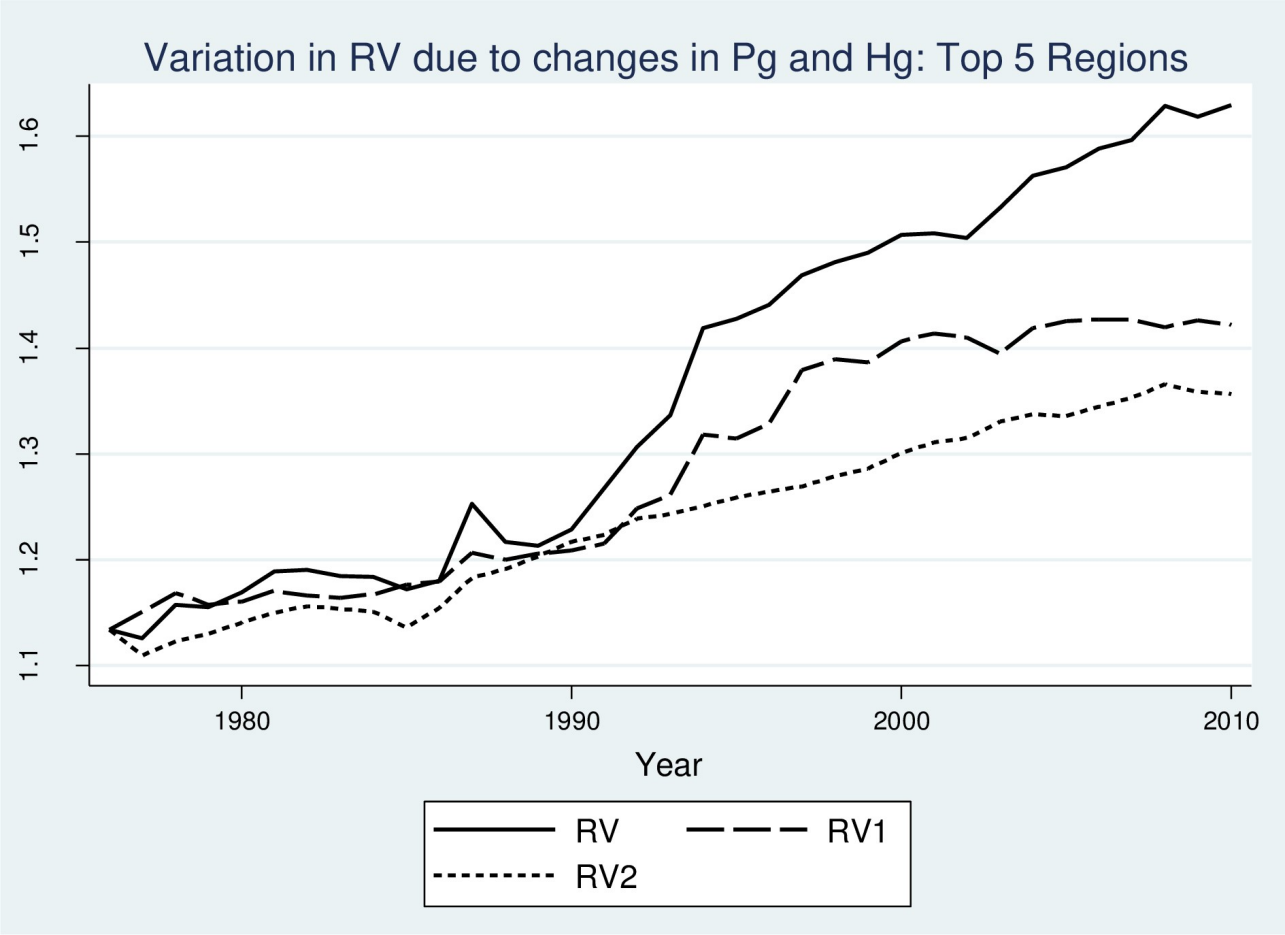
Relative importance of Pg and Hg in the growth of RV over time: Top 5 regions. Notes: RV1: Related variety measured by keeping Pg constant at the values at 1976. RV2: Related variety measured by keeping Hg constant at the values at 1976.

**Fig 9:**
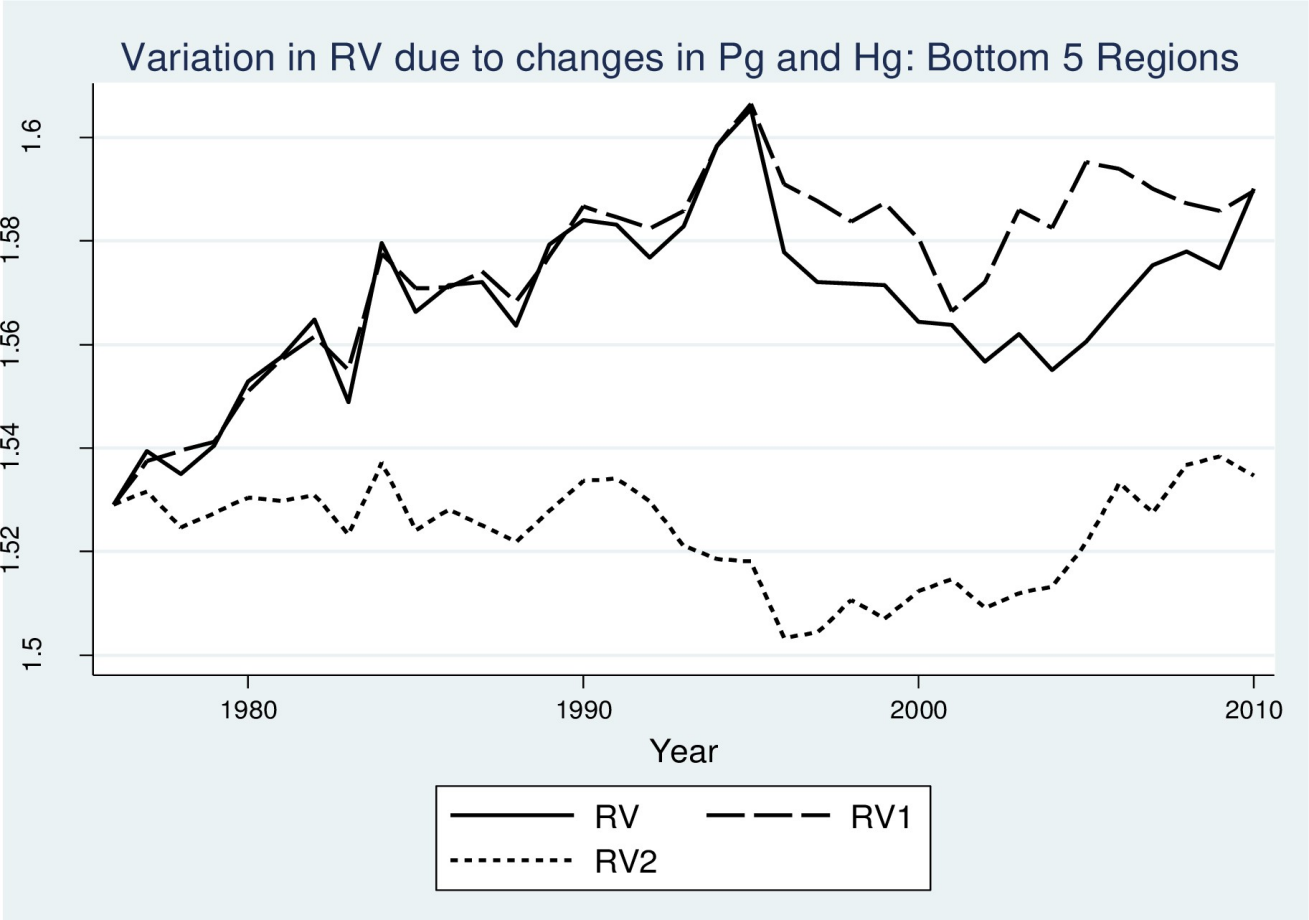
Relative importance of Pg and Hg in the growth of RV over time: Bottom 5 regions. Notes: RV1: Related variety measured by keeping Pg constant at the values at 1976. RV2: Related variety measured by keeping Hg constant at the values at 1976.

The average flows reported in Fig 7 show the overall evolution of RV in Germany. Fig 7, however, does not capture the variation of trends across regions. Since each region has its own economic structure, the evolution of industrial structure is likely to differ across regions. Moreover, the effects of Pg and Hg on the behavior of the RV index could also differ for regions that experience a significant increase in RV as compared to other regions with a less than average increase in RV. To differentiate between the evolution of variety in regions with the highest and lowest changes in RV over time, Figs 8 and 9 show the evolution of RV, RV1, and RV2 for the top 5 and bottom 5 regions in terms of changes in RV. For the sake of simplicity and ease of comparison, the Figs only show plots for median values across selected regions. For the top 5 regions, similar to Figs 7, 8 shows that both Pg and Hg have a strong positive effect on RV with the most variation in the trends arising from changes in Hg. On the contrary, Fig 9 shows that for the bottom 5 regions, Hg is the strong driving force behind RV, where Pg has no long term effect on RV. Put differently, for ‘top-performing’ regions there has been an important role for the increase in diversity at the two-digit level that is generally more difficult to achieve since regions tend to diversify in sectors that require capabilities similar to the existing ones in the region. For ‘bottom performing’ regions, until 1995, RV followed a positive trend. During this period, RV and RV1 lines are approximately parallel to each other showing that Hg is the driving force behind the increase in RV. This can also be confirmed by the relatively flat curve of RV2 for the same period which shows that there was no significant effect of Pg on the evolution of RV before 1995 in the bottom 5 regions. After 1995, RV shows a U-shaped relationship which is mostly driven by Hg, as RV1 also shows a similar trend. During this period, RV2 shows a slightly increasing trend, however, the overall effect of Pg from 1976 to 2010 in the evolution of RV, as shown by the RV2 plot, is insignificant as the initial and final positions in the graph are almost identical. From the analysis in this section, we can conclude that the driving force behind a strong increasing trend in RV is variation in industrial distribution or entropy at the three-digit level and not the overall magnitude of employment at the two-digit level which is used as a weighting variable in the calculation of RV.

#### The role of sectoral entries and exits on the increase in related variety

Related variety can increase due to a change in the number of related industries in the region or due to change in the distribution of employment across related industries. Increase in variety achieved through an increase in the shares of existing industries in the regional industry portfolio is termed as variety in *intensive margins*, while an increase in variety due to the entry of new sectors in the industrial portfolio is termed as variety in *extensive margins* [33]. An absolute increase in the number of industries in a region can be the result of entries or the net positive difference between entries and exits. If entries are approximately equal to exits, provided that the shares of new industries match the distribution of exiting industries, industrial variety will be unaffected.

The dynamics of industry entries and exits could be different for regions with the highest increase in RV as compared to the regions with the lowest increase in RV, as low RV implies that regional employment is dominated by a few industries and theoretically opportunities to enter into new industries are higher in those regions. On the contrary, if employment is relatively equally shared by existing industries, then the resulting RV will be high and it implies that there is some level of competition in each industry, therefore exits are expected to be higher due to a high level of competition. This leads to the expectation of high levels of industry dynamics in terms of entries and exits in regions with higher levels of RV.

This section aims to explore whether an increase in RV via the change in the number of industries is purely because of entries or if it is because of the net positive difference between entries and exits. The task is performed only for the top 3 and bottom 3 regions in terms of changes in RV because of the expected difference in sectoral dynamics: especially between low and high performing regions in terms of the industrial variety.

**Fig 10:**
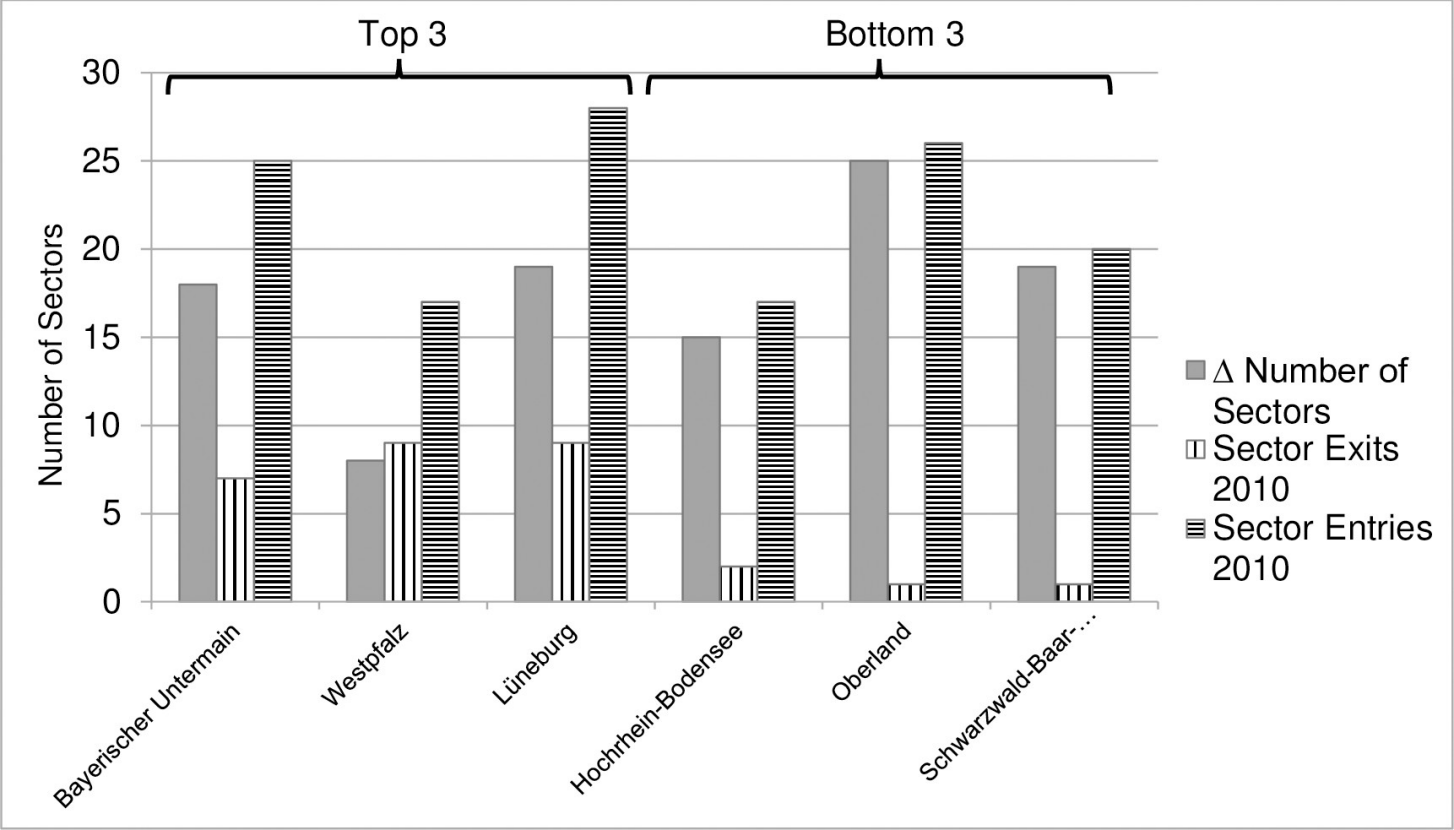
Entries and exits of industries in highest and lowest performing regions in terms of increase in RV. (Year of observation 2010, year of reference 1976). Source: Authors.

The comparison in Fig 10 and Table 2 shows that although the number of industries has increased over time for both high and low variety regions, the number of exits is different for the regions in the top and bottom group. A large number of exits in the top-performing regions could be an indication of the creative-destruction type effect where new industries take over the inefficient old ones. If new sectors replace old ones, the distribution of industries is likely to change significantly as the employment shares in existing industries may also change due to competition effects which may result in the movement towards the equalization of employment shares. A technical explanation of the difference is that entries and exits at the same time create a larger distortion than pure entries. Therefore, due to the uneven evolution of sectoral shares, diversification measures increase. Analysis of the top and bottom regions, in terms of the increase in RV, shows that sector exits in the bottom 3 regions are quite low even though sector entries are, on average, like the ones for the top 3. It appears as if new industries did not alter the previous industrial structure in the bottom group regions which might be due to an increase in the number of industries in sectors unrelated to the existing industrial portfolio, therefore, not leaving an impact of related variety measures. Unless the shares of employment absorbed by the new entering industries are large enough to change the industrial structure significantly (i.e. technically via the sectoral shares Pg, see the discussion in Section 0), it will not change the related variety noticeably. However, detailed analysis to confirm or reject these propositions is outside the scope of this study and requires further research.

**Table 2:** Entries and exits of industries in Top 3 and bottom 3 regions in terms of RV.

	Region	Change in RV 1976–2010	Number of Sectors in 1976	Number of Sectors in 2010	Change in number of sectors 1976–2010	Sector Exits (Between 2010 and 1976)	Sector Entries (Between 2010 and 1976)
Top 3	Bayerischer Untermain	0.58	137	155	18	7	25
	Westpfalz	0.52	140	148	8	9	17
	Lüneburg	0.47	126	145	19	9	28
Bottom 3	Hochrhein-Bodensee	0.056	145	160	15	2	17
	Oberland	0.053	139	164	25	1	26
	Schwarzwald-Baar-Heuberg	0.052	140	159	19	1	20

Source: Authors

#### Relation of variety with the stage of development and spatial dependence to frontiers

The natural question that arises after analysis of the evolution of variety in regions is why some regions can diversify while others are not. The question does not necessarily concern itself with the issue of causation but also implies the co-evolution of variety with other economic factors such as the level of development [4] or geographical factors such as the distance from the most diversified regions [34]. It is particularly important to study the determinants of unrelated variety as it involves riskier investments and requires a locally unavailable skill-set, as compared to related variety, which can also take place automatically through an evolutionary process due to the relatedness of required skills with the existing skill-set of a region.

Intuitively, developed regions are better able to invest in the diversified product basket, as compared to less developed regions, due to the riskiness of new investments. The non-linear relationship between development and diversification was identified by [4] who found that countries diversify at the early stages of development; however, they re-specialize after reaching a threshold level of per capita income. Moreover, also geographical proximity with highly diversified regions is expected to increase diversification in other regions as a result of knowledge spillovers and skill transfers through increased mobility of labor, trade, and entrepreneurship across borders due to reduced transportation and transaction costs [34]. Geographical proximity can also reinforce MAR, or Jacobs externalities, and reach beyond the boundaries of the region itself. Studies on knowledge spillovers highlight the importance of face-to-face contact for transfer of uncodified (tacit) knowledge, the possibility of which increases when geographic proximity is high. Moreover, entrepreneurs in the highly diversified regions are expected to make use of nearby regions for their business especially if the nearby regions are providing a favorable policy mix for the investors. As a result, nearby regions may attract locally unavailable capabilities from highly diversified regions to increase the diversification at home. One can, therefore, expect the diversification levels of regions to be high if they are in proximity to the most diversified regions.

To empirically assess the role of the stage of development and geographical proximity on the level of variety, this section uses the following empirical model:

$${(Un)Related\_Variety}_{i,t}=\beta_{0}+\beta_{1}{\frac{Emp}{Area}}_{i,t}+\beta_{2}{\left(\frac{Emp}{Area}\right)}_{i,t}^{2}+\beta_{3}{Distance}_{i,t}+\varepsilon_{i,t}$$

Where the dependent variable is either related or unrelated variety and ${\frac{Emp}{Area}}_{i,t}$ is employment in a region per square meter. Employment levels are divided by the area of the regions to minimize the scale effects. Employment levels are used to proxy the stage of regional development. The squared term of the employment level is added to test the non-linear relationship between the stage of development and diversification as identified by [4]. Following the results of [4] one would expect a positive sign for the main effect of the employment level and a negative sign for the squared employment level. Taking inspiration from [35, 36], the Distance variable is the distance of the region in question with the closest of the top 10 regions in terms of variety. In other words, each year regions are ranked in descending order according to their related and unrelated variety levels. In the next step, distance is calculated between each region in the sample and the top 10 regions in terms of variety. In the last step, the shortest of the distances calculated in the second step is taken as a value for the Distance variable for that region. Following the construction, interpretation of the variable is an inverse to the proximity variable i.e. the larger the value of the Distance variable is, the less likely a region is to have a high level of variety. Therefore, one would expect the sign of the Distance variable to be negative.

The results of the estimation are presented in Table 3. The results show strong support for the [4] results i.e. regions seem to diversify at early stages of development (measured by employment levels) and re-specialize at later stages. The result holds for both related and unrelated variety. The distance variable is found to have a negative relationship with variety, both for related and unrelated variety, confirming the expectation that regions that are distant from highly diversified regions find it difficult to diversify their product portfolio. The effect is particularly strong for unrelated variety showing that distance matters more for unrelated variety as compared to related variety as the import of locally unavailable capabilities from other regions is easier when a region is geographically proximate to highly diversified region(s).

**Table 3:** Relation of stage of development and distance to frontier with UV and RV.

Indicator	Related Variety	Related Variety	Unrelated Variety	Unrelated Variety
Employment per Sqm	1.156***	1.100***	1.689***	1.662***
	(0.135)	(0.137)	(0.143)	(0.142)
Employment per Sqm ^ 2	-1.285**	-1.240**	-4.226***	-4.313***
	(0.426)	(0.426)	(0.454)	(0.451)
Distance to the closest region with Top 10 RV		-0.205*		
		(0.0875)		
Distance to the closest region with Top 10 UV				-0.729***
				(0.126)
Constant	1.404***	1.423***	4.305***	4.361***
	(0.00675)	(0.0107)	(0.00719)	(0.0120)
R^2^	0.118	0.12	0.0663	0.0792
N	2414	2414	2414	2414

*Notes*: Dependent variable: Related or Unrelated variety; Pooled OLS regression; Standard errors in parentheses; All explanatory variables are divided by 1000 to rescale the coefficients

***, **, *, ^+^: statistically significant at 0.1%, 1%, 5% and 10%, respectively

## Conclusion

This paper investigated the long-term evolution of industrial structure in West German regions with attention to the industry diversity, which was distinguished between the overall, related, and unrelated variety. Among several industry structure measures; analysis reveals a strong and continuous increase in RV. This confirms findings in the literature [30, 31] that regions tend to diversify in related sectors. Moreover, a relatively weaker positive trend was found for overall variety while unrelated variety did not change significantly throughout. Regarding the question of the driving force behind the continuous increase in RV, several causes for the continuous increasing trend were investigated.

First, an increase in RV was investigated with the help of statistical analysis to see if the increase was dominated by the effect of changes in sectoral shares (Pg) or entropy within these sectors (Hg). Findings suggest that the entropy component (Hg) measuring variety at the three-digit industrial classification within the two-digit level is more dominant than the two-digit sectoral shares (Pg) in the overall evolution of RV. This shows that the interpretation of the RV increase in the context of an increase in variety in related sectors is viable in the case of West Germany. The dominance of Hg holds for the top 5 and bottom 5 regions in terms of increased RV as well.

To approach the question of the driving force behind the continuous increase in RV from another perspective, analysis of top and bottom regions with the most and least increase in RV was performed to explore the role of sectoral entries and exits in the dynamics of RV evolution. Findings suggest that while both the top and bottom regions experienced an increase in the total number of industries, exits were much less pronounced in the bottom regions. It suggests that an increase in variety among top regions is the result of the creative destruction type effect where new industries force inefficient old industries to leave the region. The relatively frequent entry and exit of industries ensured that the shares of industries remained relatively even and, therefore, an increase in variety for these regions was observed.

Finally, the question of the relation of variety with the stage of regional development and spatial dependence to frontiers was investigated. Concerning the relationship between development and diversification depending on the stage of regional development, the study at the regional level confirms findings by [4] who show that countries diversify at the initial stages of development, however, after a certain threshold, re-specialize by quitting inefficient sectors. Apart from that, closeness to the most diversified regions is also related to the level of diversity of a region.

While providing several new insights to the RV and UV concept, the following limitations apply to this research. First, the effect of sectoral entries and exits in shaping the variety was performed on the bases of a few top and bottom performing regions in terms of the increase in RV. In principle, this approach approximates the study of the diversification via intensive and extensive margins. Application of such a study at the aggregate level of regional industry diversification patterns could be a promising way to increase our understanding of how regional diversification takes place. Another important question for analysis of diversification would be to identify the role of start-ups and exits in the process of diversification. Are they more of related or unrelated sectors and what type of variety do they increase? Also, some macro studies have found a relationship between variety and human development such as [37, 38] and found that variety has a positive relationship with human development. Similar relationship can be analyzed at regional level. Finally, the determinants of regional diversification into related and unrelated sectors are hardly ever analyzed in the literature. Analysis in final section of results slightly touches only two dimensions of variety determinants. Nevertheless, estimations presented in that section do not aim to provide a model explaining variety types. Instead, they aim to test the relationship between the stage of development and the distance to the frontier in terms of variety. This also explains the rather low explanatory power of the models. Further work on the identification of the possible determinants of different variety types and time required for these determinants to influence variety is certainly a further avenue of research. Likewise, case studies of regions that experienced the most pronounced increase in either RV or UV might help to reveal some insights behind the structural change.
